# West Nile Virus RNA in Tissues from Donor Associated with Transmission to Organ Transplant Recipients

**DOI:** 10.3201/eid1909.130365

**Published:** 2013-09

**Authors:** Dianna M. Blau, Ingrid B. Rabe, Julu Bhatnagar, Rachel Civen, Kavita K. Trivedi, Dominique Rollin, Susan N. Hocevar, Matthew Kuehnert, J. Erin Staples, Sherif R. Zaki, Marc Fischer, the West Nile Virus Transplant-Associated Transmission Investigation Team

**Affiliations:** Centers for Disease Control and Prevention, Atlanta, Georgia, USA (D.M. Blau, J. Bhatnagar, D. Rollin, S.N. Hocevar, M. Kuehnert, S.R. Zaki);; Centers for Disease Control and Prevention, Fort Collins, Colorado, USA (I.B. Rabe, J.E. Staples, M. Fischer);; Los Angeles County Department of Public Health, Los Angeles, California, USA (R. Civen);; California Department of Public Health, Richmond, California, USA (K. Trivedi)

**Keywords:** West Nile virus, WNV, RNA, organ transplantation, tissues, viruses, solid organ transplantation, organs, transplant, immunodeficiency, organ donor, virus transmission

## Abstract

We identified West Nile virus (WNV) RNA in skin, fat, muscle, tendon, and bone marrow from a deceased donor associated with WNV transmission through solid organ transplantation. WNV could not be cultured from the RNA-positive tissues. Further studies are needed to determine if WNV can be transmitted from postmortem tissues.

West Nile virus (WNV), a mosquito-borne flavivirus, was detected in North America in 1999 and has since become endemic to the United States, where it causes annual seasonal outbreaks. An estimated 70%–80% of human WNV infections are asymptomatic (*1*). Most symptomatic persons experience acute systemic febrile illness; West Nile neurologic disease develops in <1% of infected persons but has a case-fatality rate of 9% (*2*).

Most WNV infections are acquired through bites from infected mosquitoes. However, the virus can also be transmitted by transfusion of infected blood products or by solid organ transplantation (*3*,*4*). In 6 clusters of organ transplant–transmitted WNV infections reported to public health agencies in the United States, 12 (75%) of 16 recipients were infected (*5*). Encephalitis developed in 9 (75%) of those recipients; 4 of those 9 died.

WNV transmission through tissue transplantation (i.e., skin, muscle, or connective tissues) has not been identified, and the risk for transmission by this route is not known. We evaluated tissues collected from a deceased donor who was associated with transmission of WNV through solid organ transplantation to determine if WNV RNA, viral antigen, or infectious viral particles could be detected in postmortem tissues.

## The Study

In 2011, the Centers for Disease Control and Prevention (CDC) assisted state and local health departments in an investigation of a cluster of WNV disease transmitted through solid organ transplantation (*6*). The adult male donor had a history of cerebral palsy, seizures, and blindness. He was cared for at home and had outdoor exposure in a county with known WNV activity. In late summer, he had acute onset of fever and lethargy; 2 days after symptom onset, a urinary tract infection was diagnosed, and he received oral antimicrobial drugs. The following day, he suffered cardiopulmonary arrest. After resuscitation, he remained unresponsive, and an electroencephalogram showed no cortical activity. After consent was obtained, solid organs (i.e., kidneys, lungs, and liver) and tissues (i.e., skin, fat, muscle, tendon, and bone) were procured 9 days after his illness onset. Corneas, heart valves, and vascular tissue were not procured. The donor’s organs were transplanted into 4 recipients; none of the donor tissues were transplanted.

After WNV infection was detected in 1 of the organ recipients 10 days after transplantation, the donor’s stored clinical samples (i.e., serum and spleen/lymph node homogenate) were retrospectively tested for WNV; this testing occurred within 5 weeks after transplantation. The donor’s serum sample was positive for WNV IgM, IgG, and neutralizing antibodies by serologic testing but negative for WNV RNA by nucleic acid amplification testing. WNV RNA was detected in spleen/lymph node homogenate. Subsequently, all 4 organ donor recipients were tested and had positive results for WNV RNA. Two of the recipients died of WNV infection. 

Five weeks after the donor’s death, frozen spleen/lymph node homogenate from the donor that had been used for human leukocyte antigen testing was sent from the transplant center to CDC, and initial WNV PCR testing was performed as part of the transplant-transmission investigation (*7*). Eight weeks after the donor’s death, skin samples that had been treated in cryopreservative solution containing an antibiotic and unprocessed fat, muscle, tendon, and bone samples, all of which had been stored frozen at −70°C at a tissue bank, were transferred to CDC. At CDC, the tissues remained frozen at −20°C to −70°C in individual double-wrapping and plastic bags and were handled and tested separately to reduce the risk for cross-contamination.

RNA was extracted from each 3–5 mm section of tissue by using a phenol-chloroform extraction method as described (*8*), with the following modifications. Tissue was homogenized in Buffer RLT (QIAGEN, Valencia, CA, USA) and digested with proteinase K for 15 min at 55°C; an additional 24-h digestion at 40°C was used for bone. RNA quality was ensured by amplification of housekeeping genes. RNA samples were tested by using WNV-specific reverse transcription PCR (RT-PCR) targeting the nonstructural protein 1 (NS1), capsid, and premembrane genes (*9*). Positive PCR amplicons were sequenced for confirmation. To assess for infectious virus, we injected homogenates from tissues positive by RT-PCR into Vero E6 cells; for cells with cytopathic effect, we confirmed the presence of WNV by RT-PCR, immunofluorescence, and electron microscopy. Immunohistochemical (IHC) staining for WNV was performed on RNA-positive tissues (*10*). RT-PCR was performed 25–26 weeks after the specimens were collected from the donor; virus culture and IHC staining were performed 50 weeks after specimens were collected.

WNV RNA was detected in samples from the spleen/lymph node, skin, and fat associated with the tibia bone, as well as 1 of 2 muscle specimens, 1 of 4 tendon specimens, and 1 of 2 bone marrow specimens (Table). Cytopathic effect was noted only in Vero cells injected with the spleen/lymph node homogenate; these cells were positive for WNV by RT-PCR, immunofluorescence, and electron microscopy. Cytopathic effect was not observed in Vero cells injected with skin, fat, muscle, tendon, or bone marrow. Results of IHC staining of skin, fat, muscle, and bone marrow samples were negative for WNV antigens.

**Table:** WNV in tissues from solid organ donor associated with WNV transmission to solid organ transplant recipients*

Specimens tested	Tests performed†		
	RT-PCR	Cell culture	IHC staining
Spleen/lymph node homogenate	+	+	NT
Tissues			
Skin	+	–	–
Fat, tibia	+	–	–
Tendon			
Gracilis	+	–	NT
Achilles	–	NT	NT
Semitendinosus	–	NT	NT
Tibialis	–	NT	NT
Muscle			
Attached to tibia	+	–	–
Adjacent to gracilis tendon	–	–	NT
Bone marrow			
Femur	+	–	–
Pelvis	–	NT	NT
Bone, pelvic cortical	‡	NT	NT

*WNV, West Nile virus; RT-PCR, reverse transcription PCR; IHC, immunohistochemical; +, positive; NT, not tested; –, negative. †For spleen/lymph node homogenate, fat, tendon, muscle, bone marrow, and bone, a single sample was tested for WNV RNA by RT-PCR. If positive, a second specimen was tested by RT-PCR, inoculated into Vero E6 cells, and evaluated by IHC. For skin, after initial WNV RT-PCR was positive, 3 subsequent samples were tested for WNV RNA by RT-PCR, inoculated into Vero E6 cells, and evaluated by IHC. ‡Sample inadequate for testing.

## Conclusions

We identified WNV RNA in spleen/lymph node homogenate, skin, fat, muscle, tendon, and bone marrow samples obtained postmortem from a donor associated with transmission of WNV through solid organ transplantation. WNV was isolated from the spleen/lymph node homogenate, indicating infectious virus. However, infectious virus could not be cultured, and WNV antigens were not identified by IHC staining from any of the WNV RNA-positive tissues.

Data on the detection of WNV in postmortem organs or tissues are limited. In a study published in 1954, a total of 95 patients with terminal cancer were injected intramuscularly with WNV (*11*). Among 14 patients who died within 1 month after inoculation, virus was isolated postmortem from solid organs in 11 patients and, in 1 patient each, from skin, muscle, or connective tissue. In a more recent study of 6 patients with fatal mosquito-borne WNV encephalitis, WNV RNA or antigens were variably detected in solid organ samples from all patients, and WNV antigens were identified in skin samples from 1 patient (*12*). However, 4 (67%) of these patients were severely immunocompromised transplant recipients; of the 2 immunocompetent patients, 1 had WNV RNA in brain, spleen, and kidney samples and 1 had WNV antigens only in brain samples. WNV has also been cultured from an antemortem skin biopsy sample from a patient with rare hemorrhagic manifestations of disease (*9*).

This study has several limitations. The findings are from a single donor, and thus their generalizability is uncertain. The tissues were stored frozen for almost 1 year before culture, which may have decreased the ability to isolate viable WNV, although virus was isolated from the spleen/lymph node homogenate. Several tissues that are commonly transplanted with minimal processing (e.g., corneas, heart valves, and vascular grafts) and that have been implicated in recent transmission of other viruses, such as hepatitis B and C (*13*,*14*), were not procured or tested in our study. Finally, although the specimens were stored, handled, and tested individually, false-positive results or cross-contamination cannot be completely ruled out.

Although WNV RNA was detected in unprocessed tissues obtained from the organ donor, the absence of viral antigen by IHC staining and failure to culture infectious virus from skin, muscle, and tendon suggests that the risk for WNV transmission may be lower for transplantation of these tissues than for transplantation of solid organs. Further studies are needed to determine if infectious WNV can be recovered from and possibly transmitted by transplantation of postmortem tissues and, if so, to assess the period of risk and whether tissue processing would mitigate the risk (*15*).
